# Phonological and Semantic Cues to Learning from Word-Types

**DOI:** 10.5334/labphon.39

**Published:** 2016-08-09

**Authors:** Peter Richtsmeier

**Affiliations:** Purdue University, US

## Abstract

Word-types represent the primary form of data for many models of phonological learning, and they often predict performance in psycholinguistic tasks. Word-types are often tacitly defined as phonologically unique words. Yet, an explicit test of this definition is lacking, and natural language patterning suggests that word meaning could also act as a cue to word-type status. This possibility was tested in a statistical phonotactic learning experiment in which phonological and semantic properties of word-types varied. During familiarization, the learning targets—word-medial consonant sequences—were instantiated either by four related word-types or by just one word-type (the experimental frequency factor). The expectation was that more word-types would lead participants to generalize the target sequences. Regarding semantic cues, related word-types were either associated with different referents or all with a single referent. Regarding phonological cues, related word-types differed from each other by one, two, or more phonemes. At test, participants rated novel wordforms for their similarity to the familiarization words. When participants heard four related word-types, they gave higher ratings to test words with the same consonant sequences, irrespective of the phonological and semantic manipulations. The results support the existing phonological definition of word-types.

## 1 Introduction

Word-types are abstract wordforms that are typically defined in phonological terms, that is, as being composed of a unique set of phonemes. In English, for example, *hasty*, *pessimistic*, and *esteem* are unique word-types that have the /st/ word-medial consonant sequence in common. However, these words also have unique meanings. It is commonplace for word-types to be treated as phonological entities, but it is also possible that their essential nature, or their quiddity, includes semantic properties. This study examines learning from word-types in a task in which types are commonly utilized: statistical learning of phonotactic patterns. The critical question is whether semantic cues influence how word-types support those patterns.

To better understand why semantics might play a role in phonological acquisition, consider the following hypothetical situation: A child grows up consistently exposed to multiple dialects of English, and therefore hears widely varying pronunciations of the same word. For example, she regularly hears the word *hasty* pronounced as [heɪsti], [hæɪsti], and [hʌɪsti]. With respect to learning about the phonotactic sequence /st/, is it sufficient that these pronunciations are composed of different sounds for the child to treat them as different word-types, permitting each pronunciation to serve as evidence that /st/ is a licit English consonant sequence? Or, because they share a common meaning, would she treat each pronunciation as a variation on a single word-type and wait on evidence from additional words such as *pessimistic* and *esteem* to make a generalization about /st/? To better answer this question, we first turn to previous research on word-types.

### 1.1 Previous findings on word-types

In phonological research, word-types represent the primary form of data for many grammar learning algorithms, for example, in Albright’s Minimal Generalization Learner (2009), Boersma’s Bidirectional Phonology (2011), and Hayes and Wilson’s Maximum Entropy model (2008), to name just a few. As the primary data for these grammars, word-types are responsible for establishing the grammar of a specific language. Notably, these formal models only make reference to the phonological properties of word-types.

With respect to linguistic performance, word-types have proven valuable for explaining phonological intuitions or behaviors, including past tense formation (e.g., Albright & Hayes, 2003; but cf. Moscoso del Prado Martín et al., 2004, for counterevidence) and lexical decision times (Conrad et al., 2008). Word-types also reliably predict wordlikeness judgments (Albright, 2009; Richtsmeier, 2011). In a statistical learning experiment that controlled for the total number of exposures, Richtsmeier (2011) observed that experimentally-defined high word-type frequency, but not high word-token frequency, modulated wordlikeness judgments. Adults were familiarized with word-medial consonant sequences, for example, /fp/. The sequences appeared in either one or three words, for example, just in /mæfpəm/, or in /mæfpəm/, /baɪfpəm/, and /gɪfpək/. Participants were later asked to make wordlikeness judgments for new words containing the same consonant sequences, for example, /neɪfpən/. Generalization to the novel words was only observed when participants were familiarized with multiple word-types. No generalization was observed when participants were familiarized with a single high-token-frequency word. Thus, in a direct comparison of learning from word-types and word-tokens, word-types consistently supported phonological learning in a way that word-tokens did not (cf. Albright, 2009; Bybee, 1995, for additional evidence in favor of word-types). In sum, word-types are useful entities when explaining linguistic intuitions.

Although word-types are not always explicitly mentioned, previous studies imply that they support early phonological development (Edwards et al., 2004; Ferguson & Farwell, 1975; Stoel-Gammon, 1985; etc.). In a more explicit analysis, Pierrehumbert (2003) proposed that phonological development is a process of accruing abstractions over word-types. Richtsmeier, Gerken, and Ohala (2011) tested that prediction in a study of phonotactic learning from word-types and word-tokens that controlled for the total number of exposures. Four-year-old children who heard /fp/ in the word-types /mæfpəm/, /baɪfpəm/, and /gɪfpək/ during the familiarization were subsequently more accurate to produce a new word containing /fp/, such as /neɪfpən/, than children who had only heard /mæfpəm/. In contrast, children who heard a large number of tokens of /mæfpəm/ were not more accurate when producing /neɪfpən/ than children who heard a smaller number of tokens of /mæfpəm/. The authors concluded that word-type frequency was a fundamental component of phonological generalization.

How do word-types support phonological generalization? Generally, status as a word-type is linked to phonological properties, as is the case for all of the formal grammars cited above. For example, phonological grammars reduce *sister* and *hasty* to phonological forms like /sɪstɚ/ and /heɪsti/ and look for featural or phonemic commonalities such as the co-occurrence of /st/. Importantly, most grammars treat /sɪstɚ/ and /heɪsti/ as equivalent pieces of evidence for the status of /st/ because they are both word-types. The fact that *sister* has a greater word-token frequency than *hasty* in the Corpus of Contemporary American English (Davies, 2008) does not influence how these grammars peform.

However, *sister* and *hasty* also have unique meanings, so word-type status is signaled by different meanings as well as by different phonological forms. For a learner, if the change in meaning from female sibling (*sister*) to precipitous behavior (*hasty*) is clear, then the learner can track the change in word-types, even in the presence of degraded or misleading acoustics. It is therefore possible that their meanings allow *sister* and *hasty* to act as cues for the phonological status of /st/. From the perspective of language development, we may ask whether both the phonological and semantic properties of word-types support phonological learning.

### 1.2 Previous research linking semantics and phonology

Within the existing literature, the influence of semantics on phonology is unclear. Some studies suggest semantics can play a role in determining phonological status, while other studies do not. Equally challenging is the fact that different studies have approached different aspects of phonology. Peperkamp and Dupoux (2007) had adult participants learn allophonic alternations that depended on a preceding determiner. An allophonic alternation is a sound change within a word that is conditioned by the word’s surrounding phonological environment. In Peperkamp and Dupoux’s study, the /k/ onset in *kinel* was conditioned by the determiner *nel*, as in *nel kinel*, but the /g/ onset in *ginel* followed *ra*, as in *ra ginel*. Peperkamp and Dupoux rightly point out that allophonic alternations are learnable based on both phonological and semantic cues. For example, participants could learn the alternation by tracking the phonological context that conditions the alternation, that is, whether the preceding determiner ends with a vowel or a consonant. However, if the surface forms *ginel* and *kinel* are both associated with the same visual referent, then participants could also use this semantic cue to learn the alternation.

Participants in this study were trained on velar stop (/g/ ~ /k/), labial stop (/b/ ~ /p/), or unnatural (/g/ ~ /f/) alternations. They were subsequently tested on the training word pairs, on novel pairs using the trained velar and labial alternations, and on novel pairs with an untrained alveolar alternation (/d/ ~ /t/). When they had visual referents for both allophonic variants, that is, when semantics provided a robust cue for the alternation, participants successfully applied the alternation to both old and novel word pairs. When just one form of the alternation was accompanied by semantic cues, that is, when semantic cues were uninformative about the alternation, they were not successful. No participant group applied the alternation to the untrained alveolar pair. The results indicate that semantic cues support learning of an allophonic alternation, even when the alternation involves phonologically unrelated sounds. However, participants only applied the alternation to the familiarized sound pairs, indicating that phonological generalization was absent.

Several studies (Creel, 2012; Swingley & Aslin, 2000; Yeung & Werker, 2009) have looked at whether semantic cues influence the interpretation or acquisition of phonological contrasts, such as the difference between /b/ and /p/. Yeung and Werker (2009) studied phonetic categorization in 9-month-old infants. Without training, 9-month-old infants acquiring English were unable to distinguish a Hindi dental-retroflex stop contrast ([ɖa] vs. [d̪a]). When infants were trained on the contrast, visual referents appeared to be critical to learning. That is, infants learned the contrast when it was supported by consistent pairings of visual referents to the two categories. They did not appear to learn the contrast when the visual referents were inconsistent—or assigned to both categories at the same time.

Semantic cues have also influenced performance in categorization tasks with odd pronunciations. Creel (2012) found that preschoolers often select known referents such as ‘fish’ when searching for a referent for odd pronunciations such as *feesh*, /fiʃ/. Similarly, Swingley and Aslin (2000) found that 18–23-month-old infants look at a known referent such as ‘baby’ if they hear an odd pronunciation such as *vaby*, /veɪbi/, although they look less reliably. The implication of the Yeung and Werker (2009), Creel (2012), and Swingley and Aslin (2000) studies is that semantics may influence the interpretation of acoustic differences near phonological borders. However, it is not clear how similar these tasks are to type-based learning or to phonological generalization.

Other studies suggest that semantics has little or no role in phonological development simply because meaning is absent. Many artificial language learning studies report phonological learning effects without containing any semantic component, that is, using only auditory or written materials (e.g., Finley, 2013; Goldrick & Larson, 2008; etc.). These studies focus solely on phonological learning, but they suggest that semantic properties are not necessary for learning in the phonological domain.

In sum, previous research on the link between semantics and phonology has involved a wide variety of methods and has included disparate phonological learning targets, and so it remains unclear what contribution semantics might make to learning from word-types. As such the present study is unique in examining the relationship between semantics and phonological learning. Here, a basic semantic property—synchronous referent-wordform pairing—is examined in a statistical learning task, specifically, learning about word-medial phonotactic sequences. The goal was to determine whether semantic cues could modulate an anticipated learning effect. As discussed above, the existing literature does not clearly predict whether semantics plays a role in type-based learning, so two rough hypotheses will be considered. Hypothesis 1: Semantics does modulate phonotactic learning, with the semantic influence depending on whether acoustically varying forms have the same or different referents. This hypothesis is inspired by the work by Peperkamp and Dupoux (2007), Creel (2012), and Yeung and Werker (2009), but differences in methods and phonological targets across these studies limit their applicability. Therefore, Hypothesis 1 is most clearly motivated by the close association of semantic properties to word-type status observable in the natural world. Hypothesis 2: Semantics will not modulate phonotactic learning. Hypothesis 2 is motivated by the existing definition of word-types as purely phonological, but also by consistent phonological generalizations observed in statistical learning studies without a semantic component (Finley, 2013; Goldrick & Larson, 2008).

The experiment also varied the degree of phonological similarity between words. This was in part to simulate the hypothetical example of a child hearing multiple pronunciations of *hasty*, and as such, to create an experimental scenario in which phonologically different forms might be treated as the same. Manipulating phonological similarity also created an opportunity to explore the importance of phonological variability for supporting phonotactic generalizations. Phonological learning algorithms treat all word-types equivalently (Albright, 2009; Boersma, 2011; Hayes & Wilson, 2008), but perhaps type-based learning is more effective when the word-types are more dissimilar. As such, the phonological dimension of word-type frequency is also worth exploring empirically.

## 2 Introduction to Method

This study was an investigation of whether learning from word-types depends on the words’ phonological properties, semantic properties, or both. A statistical phonotactic learning task was chosen to examine learning from word-types. The learning targets were word-medial consonant sequences that could be learned during a brief familiarization. Participants were familiarized with a set of words containing the targets, like the /st/ in mɪstəm/, /meɪstəm /, /mεstəm/, and /mæstəm/. They were then presented new wordforms containing the targets, for example, /nastək/, and they were asked to rate how much the new wordforms sounded like the familiarization words. If participants were influenced by the familiarization, they should have generalized from familiarization words to test wordforms. That is, participants who were familiarized with four words containing /st/ should rate /nastə*k*/ as relatively wordlike.

The experiment was conducted under the assumption that phonotactic learning is consistently guided by generalizations made over word-types. The design does not address related hypotheses, for example, that phonotactic sequences can instead be learned from word-tokens. For readers interested in this topic, there are extended discussions available in Albright (2009), Bybee (1995), and Richtsmeier (2011). The present study was limited in its focus to the semantic and phonological properties of word-types, so the design closely followed Richtsmeier (2011), in which learning effects were attributable to word-type frequency. As such, the design allowed for the expectations that (1) learning from word-types would occur and (2) phonological and semantic influences on that learning could be explored.

Some operational definitions may be helpful. Within the experiment, the term *words* refers to novel words that appeared in the initial familiarization and that were described to participants as Martian animal names. That is, *words* had both phonological and semantic properties. In contrast, the term *wordforms* refers only to the phonological properties of a novel word or to an experimental item presented without a referent, as was the case for test items. All experimental words shared a CVCCVC shape. Words varied in stress patterns, with some having word-initial stress and others having word-final stress. For example, /ˈmis.təm/ has word-initial stress and /mis.ˈtʌm/ has word-final stress.

A division between *word-types* and *word-tokens* is assumed, with word-types varying acoustically such that each type is composed of a unique set of phonemes. For example, /mɪstəm/ and /mæstəm/ contain different initial vowel phonemes and so are treated as different word-types. Word-tokens refer either to repetitions of the same acoustic token or to acoustic variations that do not result in a phonemic change, for example, different talkers’ productions of /mæstəm/. Of course, the acoustic distinction between word-types and word-tokens is not well understood. These differences are touched on briefly by the present experiments, and we return to the type/token distinction in the general discussion.

Following Richtsmeier (2011), in place of the term learning, the term *extraction* describes the behavior of participants during the test phase. Extraction refers to participants’ sensitivity to the experimental manipulations of the target sequences as measured by changes to their wordlikeness ratings, and it may reflect both priming of existing knowledge (Goldinger, 1996) as well as the acquisition of new knowledge (Pierrehumbert, 2003).

All procedures were performed in compliance with relevant laws and institutional guidelines. The study was approved by the supporting university’s institutional review board.

## 3 Method

### 3.1 Participants

One hundred and seventy-nine undergraduates at a Midwestern university completed the experiment. All participants were native English speakers with less than two years of formal training in a foreign language, and they reported having no prior history of speech, language, hearing, or cognitive difficulties. Participants completed the study for course credit or for monetary compensation. Participants were assigned to conditions semirandomly, with an equivalent number of men and women placed in the between-subjects groups.

### 3.2 Design and materials

The design was similar to other statistical language learning studies (e.g., Finley, 2013; Goldrick & Larson, 2008; Richtsmeier, 2011). The experiment began with a familiarization phase followed by a test phase. All of the experimental manipulations occurred during the familiarization and can be understood as manipulations of the familiarization words. These manipulations comprised four experimental factors: English frequency, experimental frequency, phonological overlap, and semantics. To assist with evaluation of the design, Table 1 provides a brief description of each experimental factor.

#### 3.2.1 Target word-medial consonant sequences

Eight word-medial consonant sequences were selected as extraction targets. To add a level of control over whole-word phonotactics, the same word frame was used for two sequences (one high and one low English frequency). For example, the frame /ˈmɪ__əm/ was used for /ˈmɪf.pəm/ and /ˈmɪs.təm/. A given frame was always assigned to the same two sequences: /fp/ and /st/, /mk/ and /mp/, /pk/ and /kt/, and /ʃp/ and /sp/.

#### 3.2.2 English frequency

Four sequences were infrequent in English (/fp/, /mk/, /pk/, and /ʃp/); four were frequent (/st/, /mp/, /kt/, and /sp/; cf. Vitevitch & Luce, 2004, for frequency calculations). Participants were familiarized with all eight sequences, making English frequency a within-subjects factor.

Higher ratings were expected for test wordforms containing low English frequency sequences, a reversal of what is typically obtained in novel word rating tasks. The familiarization words were described to participants as Martian, rather than English, and so the instructions may reverse expectations based on English frequencies. This hypothesis is expanded on in the General Discussion.

#### 3.2.3 Experimental frequency

The frequency of word-medial sequences varied within the experiment, and some sequences appeared in more familiarization words. Half of the participants heard a given sequence in the low experimental frequency condition, or in a single familiarization word repeated four times (e.g., four identical tokens of /ˈmɪs.təm/ from a single speaker). The other participants heard that sequence in the high experimental frequency condition, in four phonologically different familiarization words each repeated four times (e.g., four tokens each of /ˈmɪs.təm/, /ˈmeɪs.təm /, /ˈmεs.təm/, and /ˈmæs.təm/; each token was produced by a different speaker). Effects of word-type frequency were inferred based on the relative difference between high and low experimental frequency conditions.

Experimental frequency was manipulated within subjects, and each participant was familiarized with four high and four low experimental frequency sequences. Higher ratings were expected for high experimental frequency sequences (4 words × 4 tokens = 16 exposures) compared to low experimental frequency sequences (1 word × 4 tokens = 4 exposures). However, the purpose of the experiment was not simply to establish experimental frequency effects, which have been shown elsewhere (e.g., Gerken & Bollt, 2008; Richtsmeier, 2011; Richtsmeier et al., 2011). Rather, the experimental frequency effect was expected and thereby provided a baseline from which we could address the contribution of the phonological and semantic factors.

#### 3.2.4 Phonological variation

Three word lists were created: low, moderate, and high phonological variation lists. The lists varied the number of overlapping phonemes across related word-types. As discussed in the Introduction, it is unclear whether forms as similar as [heɪsti], [hæɪsti], and [hʌɪsti] function as tokens of *hasty* or qualify as unique word-types. The purpose of manipulating phonological variation was to better understand the degree of sound variation sufficient to allow for phonotactic generalization. Phonological variation was minimal in the low variability list, more substantial in the moderate variability list, and greatest in the high variability list.

Each participant was familiarized with a single list, so phonological variability was a between-subjects factor. Unlike the English and experimental frequency effects, which are well established in this experimental paradigm, the phonological variability factor is more novel, and expectations for its effect are less clear. As such a between-subjects design was chosen for this factor. The between-subjects design was intended to avoid carryover effects and simplify interpretation (e.g., Keppel & Wickens, 2004). Predictions related to the phonological variation factor are presented in Section 3.2.7, High phonological variability familiarization word list.

#### 3.2.5 Low phonological variability familiarization word list

The low variability list is presented in Table 2.^1^ Considering related familiarization words, or the words containing the same target sequence, only the first vowel varied, and consonants did not vary at all. Furthermore, the initial vowels were either all front vowels, as was the case for the /st/ words: /ˈmɪs.təm/, /ˈmeɪs.təm /, /ˈmɛs.təm/, and /ˈmæs.təm/; or were all back vowels, as was the case for the /kt/ words: /ˈbuk.təs/, /ˈbʌk.təs/, /ˈbak. təs/, and /ˈbaʊk.təs/.

Research by Van Ooijen and colleagues suggests that vowels are less critical for establishing phonological contrast (e.g., Cutler et al., 2000). As such, the low variability list represents a condition in which related word-types could be treated as varying pronunciations of the same word, and generalization of the target sequences might be limited. At the same time, the limitations of vowel differences may allow other factors, such as semantics, to play a more prominent role.

The low variability list also differed slightly from the other lists because the low experimental frequency words were not used as one of the four high experimental frequency words. However, participants still only heard one low experimental frequency word versus four high experimental frequency words, so this difference was not expected to influence the results.

#### 3.2.6 Moderate phonological variability familiarization word list

In the moderate variability list, both the first and second vowels varied, as did the stress pattern, as can be seen for the /st/ words /mis.ˈtʌm/, /məs.ˈtʌm/, /ˈmɛs.təm/, and /ˈmæs.təm/. The second vowel varied as a function of word stress. Two words carried final stress, for example, /mis.ˈtʌm/ and /məs.ˈtʌm/. The final stressed vowel was always /ʌ/ so that it differed minimally from the schwa in the words with initial stress, for example, /ˈmɛs.təm/ and /ˈmæs.təm/. Variation of the first and second vowels in the moderate variability list was intended to increase the salience of the word-medial sequence without changing any consonants. Stress pattern was not intended to be an independent factor, however. Rather, it was meant to provide acoustic variation while allowing for the possibility that related word-types could be treated as varying pronunciations of the same word.

#### 3.2.7 High phonological variability familiarization word list

In the high variability list, most phonemes surrounding the word-medial consonant sequences changed across the four related words, for example, /mis.ˈtʌm/, /məs.ˈtʌm/, /ˈʃeɪs.tək/, and /ˈbaɪs.təm/, but only the word-medial consonants repeated across all four words. Two of the four words, the two with word-final stress, also appeared in the moderate variability list. The other two words had initial stress with initial CVs and final VCs that differed from the words with word-final stress. Compared with previous lists, the high variability list was an attempt to maximize phonological differences among related words, and there was no expectation that participants might equate them.

Based on the definition of word-types derived from existing models of phonological learning (Albright, 2009; Boersma, 2011; Hayes & Wilson, 2008), all three familiarization lists contain phonologically unique wordforms and should therefore support phonological extraction. However, if extraction is dependent on relative similarity, a property that is not considered by those models, then extraction may not occur for the less variable lists, or perhaps extraction may be more robust in the more variable lists. If phonological variability influences how participants treat the familiarization words, we should predict an experimental frequency × phonological variability interaction, or an experimental frequency effect that increases with greater phonological variability.

#### 3.2.8 Semantics

**Figure 1:**
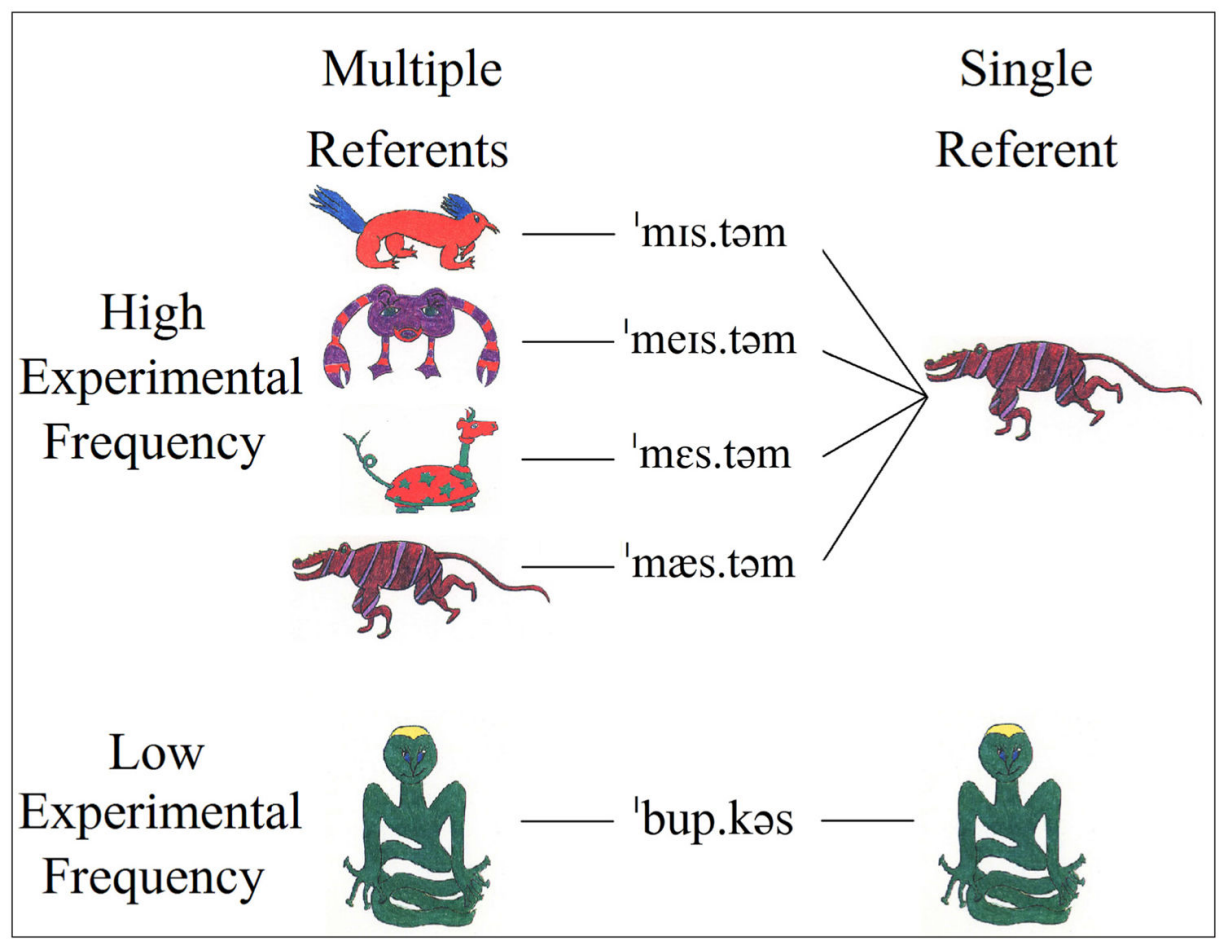
A visual depiction of the multiple referents and single referent conditions for the /st/ words in the low variability familiarization word list. Note that the manipulation only affects referent-to-wordform mappings in the high experimental frequency condition. In the low experimental frequency condition, there is always a single referent mapped to a single wordform.

A visual depiction of the semantics factor and referent-to-wordform mappings is given in Figure 1. Like the phonological variability factor, the semantics factor was manipulated across rather than within subjects to avoid carryover effects and simplify interpretation (Keppel & Wickens, 2004).

The multiple referents condition is represented on the left side of Figure 1. Participants in the multiple referents group saw a different make-believe animal for each high experimental frequency word (containing the same word-medial sequence). That is, there would be four visual referents, one each, for /ˈmɪs.təm/, /ˈmeɪs.təm /, /ˈmɛs.təm/, and /ˈmæs.təm/. The single referent condition is represented on the right side of Figure 1. Participants in the single referent group saw the same make-believe animal for each high experimental frequency word containing the same word-medial sequence. That is, there was a single visual referent mapped to /ˈmɪs.təm/, /ˈmeɪs.təm /, /ˈmɛs.təm/, and /ˈmæs.təm/. As can be seen at the bottom of Figure 1, low experimental frequency words were always paired with one make-believe animal. Because only one wordform ever appeared in the low experimental frequency condition, the semantics factor was not relevant to it.

In the single referent condition, referent-to-wordform mappings suggest that related wordforms are varying pronunciations of the same word. As such, the single referent condition could attenuate or nullify the experimental frequency effect. In the multiple referents condition, referent-to-wordform mappings suggest that related wordforms are different words. As such, the multiple referents condition should lead to a robust experimental frequency effect. If semantics does influence how participants treat the familiarization words, we should predict an experimental frequency × semantics interaction, or an experimental frequency effect only in the multiple referents condition. If the interaction also depends on the phonological similarity of the familiarization words, however, then the frequency × semantics interaction might occur for some lists but not all, for example, only for the low variability list. Such a possibility was explored in planned analyses of the experimental frequency × semantics interaction for each level of phonological variability.

#### 3.2.9 Test word list

The test wordforms are presented in Table 3. Participants did not hear the test wordforms prior to rating them, but the test wordforms contained the same sequences that participants heard during the familiarization. The sequence /st/ appeared in the test wordforms /ˈgʌs.tək/, /ˈnas.tək/, and /ˈtaʊs.tən/. All test wordforms had word-initial stress, which was common to all familiarization lists. Wordforms were produced by one of two speakers that were not heard during the familiarization, with each test speaker assigned to different lists (see below). The order that participants heard and rated the test wordforms was randomized for each participant.

#### 3.2.10 Recording of word lists

The familiarization and test words were recorded by 10 adult female speakers of a Midwestern dialect of North American English. Recordings were made in a sound booth to ensure high quality. To ensure phonetic consistency, such as appropriate stress, the speakers produced three tokens of each word immediately after a model provided by the author. A single token of each word from each speaker was then chosen for the experiment and its peak intensity was scaled to a standard value using Praat software (www.praat.org; 70 dB standard).

### 3.3 Procedure

Participants were told that they would start by learning the invented language “Martian” and the names of Martian animals; later they would rate “potential Martian animal names” for similarity to Martian. The experiment was broken up into two blocks, with each block subdivided into a familiarization block and a test block. Each block featured four of the eight target sequences. Two of the four sequences in each block were high experimental frequency (2 sequences × 4 words × 4 repetitions = 32 tokens); two were low experimental frequency (2 sequences × 1 word × 4 repetitions = 8 tokens). Different talkers produced the eight familiarization words within a familiarization block, one talker per word. Participants heard a total of 40 word tokens during each familiarization block, and each block lasted less than two minutes. The order of presentation was randomized by Paradigm experimental software (http://www.paradigmexperiments.com/).

Following each familiarization phase, participants rated how much the test wordforms sounded like the Martian animal names from the familiarization. Ratings were made on a 1–7 Likert scale, where 1 meant “definitely not a Martian animal name”, 4 meant “neutral”, and 7 meant “a great Martian animal name”. Intervening numbers were also given labels for reference. Ratings were collected for three test wordforms per target sequence, resulting in 12 ratings for each test block (24 total ratings per participant). Participants typically needed less than 2 minutes to complete each test block. The order of presentation of test wordforms was also randomized.

Eight different orderings of each of the three familiarization word lists were created. This resulted in a total of 24 orderings of the experiment. Regarding the semantics factor, four orderings of each list used multiple referents, and four used single referents. To counterbalance experimental frequencies, four orderings of each list (two in each semantic condition) assigned /kt/, /st/, /pk/, and /fp/ to the high experimental frequency condition and /mp/, /sp/, /mk/, and /ʃp/ to the low experimental frequency condition. The other four orderings reversed this assignment. High and low English frequency sequences were divided evenly across the high and low experimental frequency conditions. Additionally, two orderings were created that varied the order in which target sequences were presented. For example, one ordering presented /kt/, /st/, /mk/, and /ʃp/ in the first experimental block and /pk/, /fp/, /mp/, and /sp/ in the second experimental block. Another ordering reversed that order. Four orderings of each list—independent of the phonological variability and semantics factors—are provided in Table 4 below.

### 3.4 Analysis

Ratings were automatically recorded to a spreadsheet by Paradigm software. The data were then scanned according to pre-established criteria to filter out results from inattentive participants. Data were excluded if the participants made the same rating five or more times in a row (*N* = 15), if they made four or more ratings less than 300 ms after the end of the test wordform (*N* = 8), or if they had repetitive ratings and they made four or more ratings less than 300 ms after the offset of the test wordform (*N* = 10). Six participants from the moderate variability condition were added to the low variability condition after it was determined that, due to experimenter error, the lists that they heard were low variability lists. The result was a total of 66 participants in the low variability condition (35 single referent), 52 participants in the moderate variability condition (23 single referent), and 61 participants in the high variability condition (30 single referent). All responses made at least 300 ms from the offset of the test wordform were entered into the statistical analyses (52 responses removed).

### 3.5 Results and Discussion

Ratings were averaged across test words to derive four data points per participant, one for each of the within-subjects conditions: high English + high experimental; high English + low experimental; low English + high experimental; and low English + low experimental. Rating means (grand *M* = 4.25, SD = .62) were then entered into a 2 English frequency × 2 experimental frequency × 3 phonological variability × 2 semantics mixed design ANOVA. For all analyses, the *r* statistic is reported as a measure of effect size (Cohen, 1988; Field, 2009). An *r-*value near .1 is a small effect size, near .3 is a medium effect size, and at .5 or above is a large effect size.

**Figure 2:**
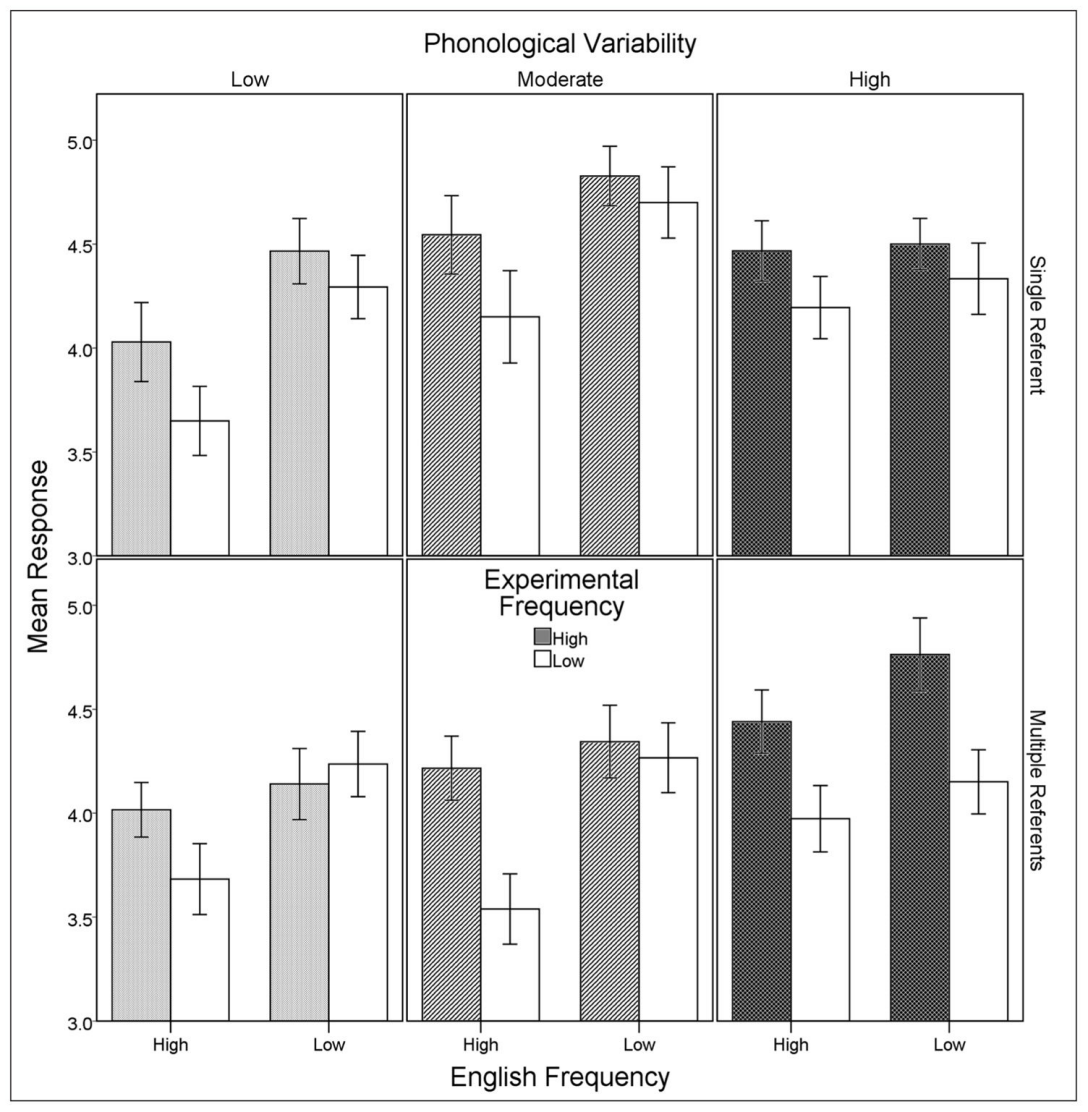
Rating means across the phonological variability factor (panel columns), semantics (panel rows), English frequency (bar groups), and experimental frequency (bar shading). Different fill patterns are used for each level of phonological variability to allow for comparison of the factor across figures. Error bars reflect mean standard errors.

**Figure 3:**
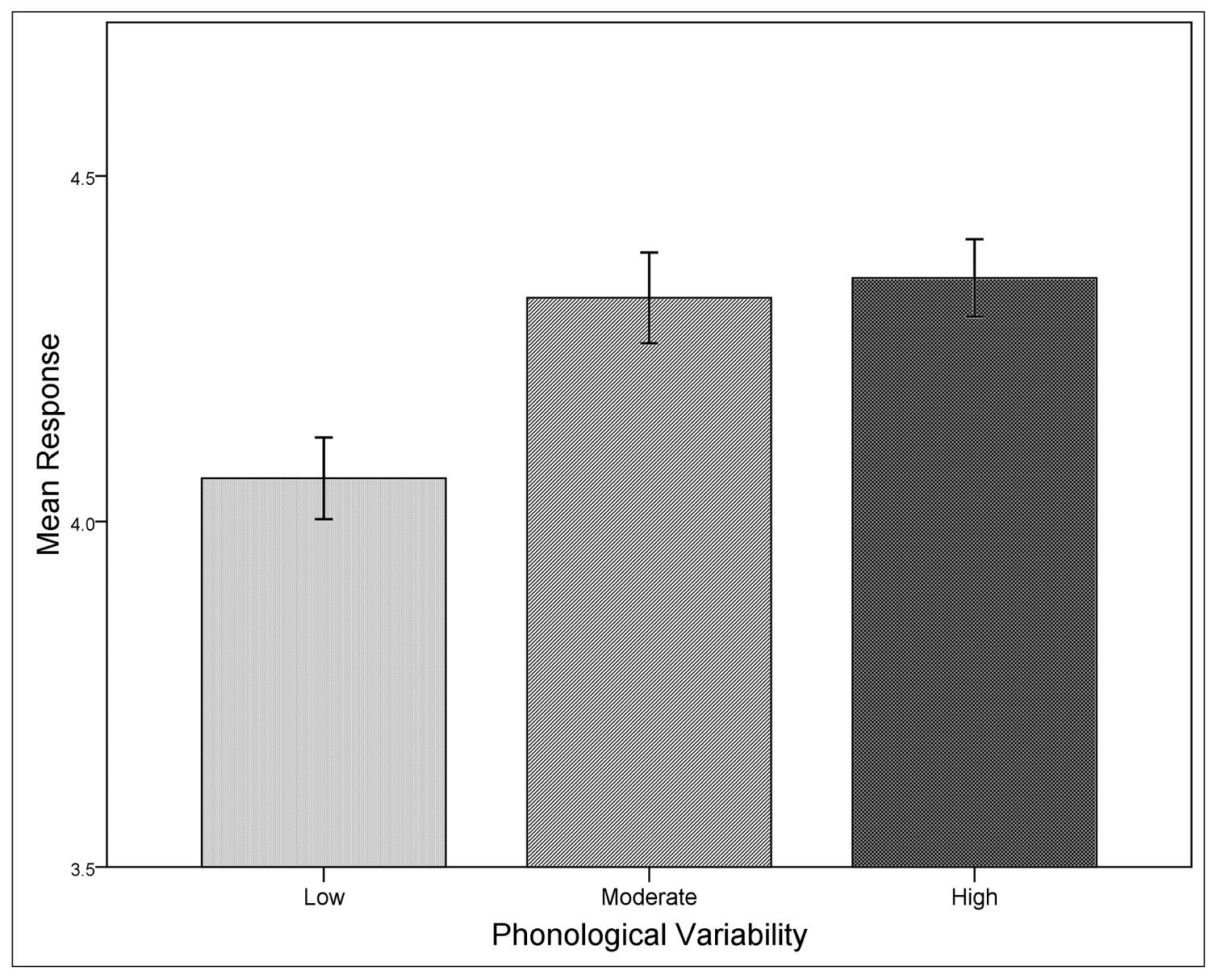
Rating means for the three phonological variability conditions. Error bars reflect mean standard errors.

A graph of the results that shows means for all conditions is given in Figure 2. There was a significant effect of English frequency, *F*(1, 173) = 43.27, *p* < .001, *r* = .20, with higher ratings being given to words containing low English frequency sequences, *M* = 4.42, than to words containing high English frequency sequences, *M* = 4.08. Experimental frequency was also significant, *F*(1, 173) = 26.34, *p* < .001, *r* = .13, and higher ratings were given to wordforms whose sequences appeared in multiple familiarization words, *M* = 4.39, compared to sequences that appeared in one familiarization word, *M* = 4.10. The semantics factor was significant, *F*(1, 173) = 5.65, *p* = .019, *r* = .03, attributable to higher ratings in the single referent condition, *M =* 4.36, compared to the multiple referents condition, *M* = 4.14. Finally, there was a nonsignificant trend observed for phonological variability, *F*(2, 173) = 2.54, *p* = .082, *r* = .01. Means for the three levels of variability are as follows: low phonological variability *M* = 4.11, moderate phonological variability *M* = 4.29, high phonological variability *M* = 4.34. Thus, ratings increased numerically when phonological variability also increased. Mean ratings by phonological variability condition are plotted in Figure 3 below.

**Figure 4:**
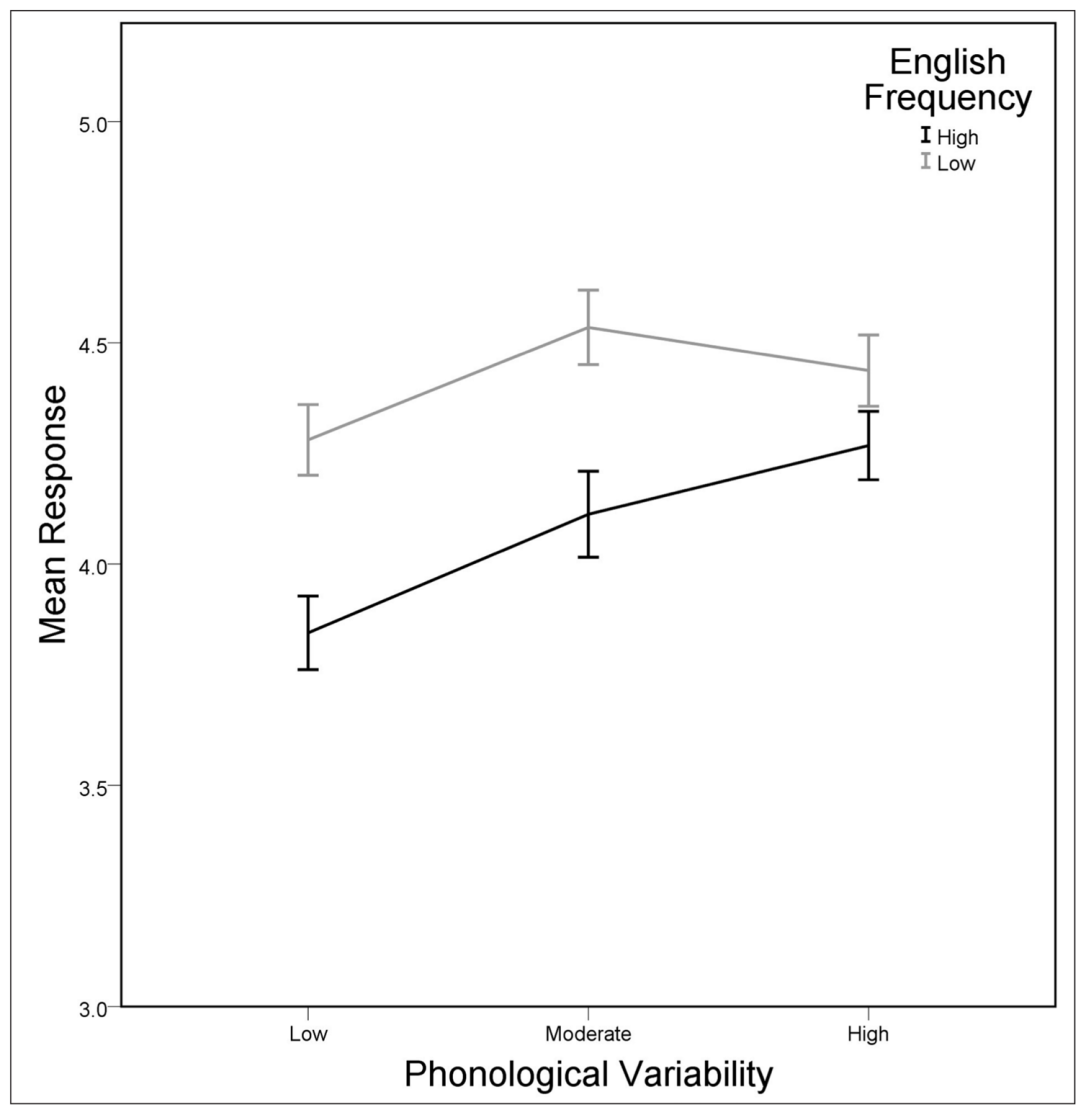
The effect of English frequency for each level of phonological variability. There are large differences between high and low English frequency sequences for the low and moderate variability lists compared to the high variability list. Note the higher ratings for words with low English frequency sequences. Error bars reflect mean standard errors.

There were two significant interactions (*p*s > .10 for all other interactions), both involving English frequency. First, the English frequency × phonological variability interaction was significant, *F*(2, 173) = 3.09, *p* = .048, *r* = .02. Considering the English frequency effect for each level of phonological variability, English frequency was significant for the low phonological variability words, *F*(1, 65) = 34.32, *p* < .001, *r* = .35, and for the moderate phonological variability words, *F*(1, 51) = 18.68, *p* < .001, *r* = .27. In contrast, only a nonsignificant trend was observed for the high phonological variability words, *F*(1, 60) = 2.92, *p* = .092, *r* = .05. Thus, the interaction is the result of significant and large effects of English frequency in the low and moderate phonological variability conditions but not in the high phonological variability condition. The trend for English frequency in the high phonological variability condition was nevertheless in the expected direction of higher ratings for low English frequency sequences. A graph of English frequency for each level of phonological variability is presented in Figure 4 and bears out the difference in magnitude of the English frequency effect across the three levels of phonological variability.

**Figure 5:**
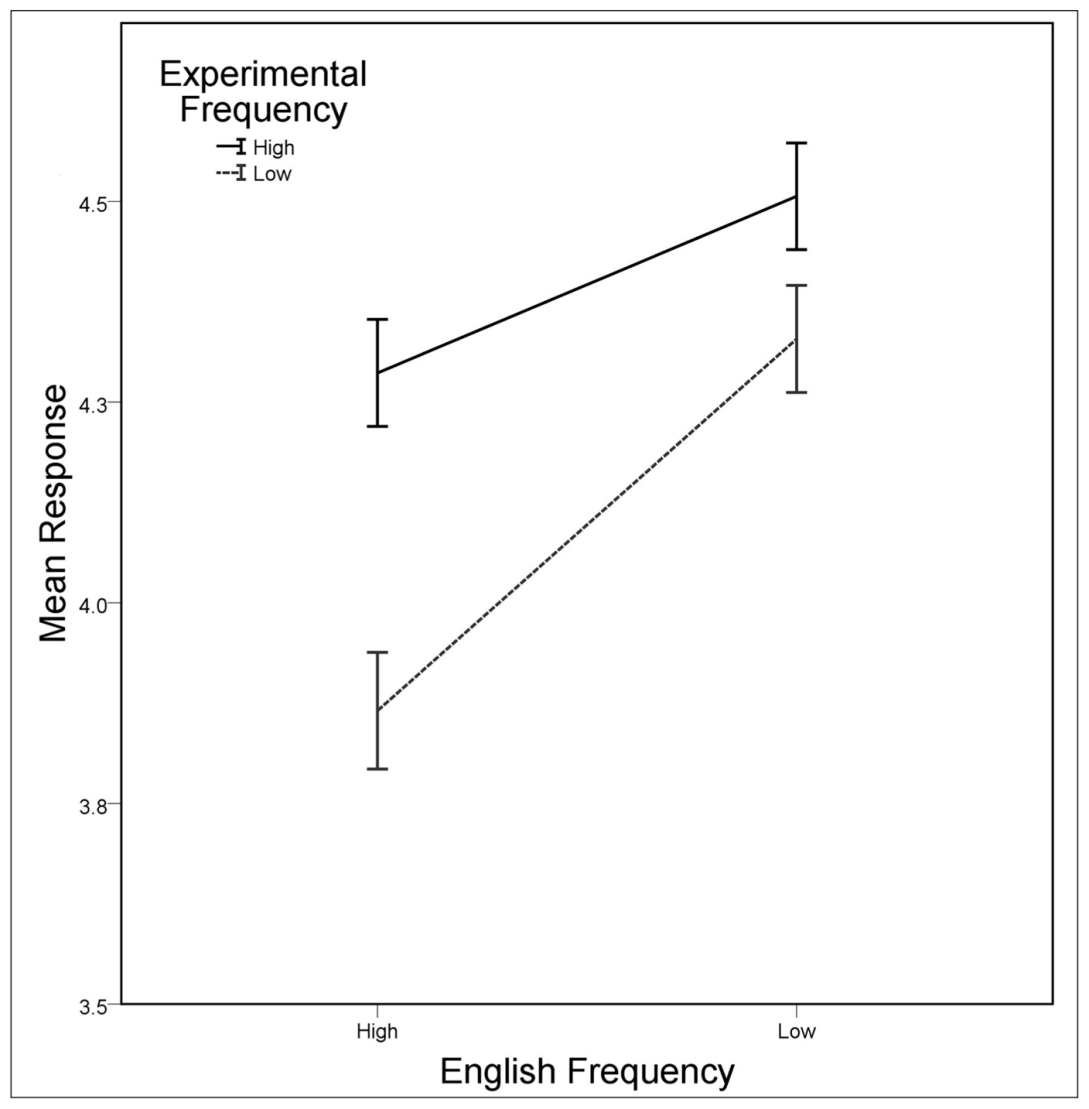
Experimental frequency at each level of English frequency. Higher ratings were consistently given for high experimental frequency sequences, but the effect was greater for high English frequency sequences than for low English frequency sequences. Error bars reflect mean standard errors.

There was also a significant English frequency × experimental frequency interaction, *F*(1, 173) = 4.01, *p* = .047, *r* = .02. Experimental frequency had a more robust effect for words with high English frequency sequences, *F*(1, 178) = 27.27, *p* < .001, *r* = .13, compared to the experimental frequency effect for low English frequency sequences, *F*(1, 178) = 4.53, *p* = .035, *r* = .02. Low English frequency sequences were generally less amenable to extraction effects. This can be seen in Figure 5, where the difference between high and low experimental frequency conditions is much larger for high English frequency.

As discussed above, if semantics played a role in driving sequence extraction, a semantics × experimental frequency interaction was expected, although we considered the possibility that the interaction might also depend on the level of phonological variability. The semantics factor did not interact with any other factor in the main analysis, but planned analyses of semantics and experimental frequency were conducted at each level of phonological variability to more fully assess the prediction.

Rating means from each level of phonological variability were entered into planned 2-way ANOVAs with the experimental frequency and semantics factors. For the low phonological variability list, experimental frequency was significant, *F*(1, 64) = 4.02, *p* = .049, *r* = .06, but semantics (*p* = .249) and the interaction (*p* = .508) were not significant. For the moderate phonological variability list, experimental frequency was significant, *F*(1, 50) = 9.17, *p* = .004, *r* = .15, as was semantics, *F*(1, 50) = 5.72, *p* = .021, *r* = .10, but the interaction was not (*p* = .906). Finally, for the high phonological variability list, experimental frequency was significant, *F*(1, 60) = 13.87, *p* > .001, *r* = .19, but the main effect of semantics (*p* = .716) and the interaction (*p* = .117) were not significant. Thus, the planned analyses reinforce the main analysis, indicating that participants extracted the targets regardless of how referents were assigned to wordforms.

## 4 General discussion

Of primary interest in these experiments were the relative contributions of phonological and semantic factors to phonotactic learning, and by extension, to the quiddity of the word-type. Word-types are typically defined by their phonological characteristics, that is, by being composed of a unique set of phonemes. In natural human interaction, however, word-types are accompanied by unique referents. This observation led to a question: Can semantics act as a cue to word-type status, and thereby to phonotactic learning? To test this possibility, the semantic and phonological properties of word-types were manipulated to gauge which cues most consistently contributed to a statistical learning effect. Learning was observed regardless of the semantic and phonological manipulations, a finding that favors the existing phonological definition of word-types, but it is worth considering each of the four experimental factors in turn.

### 4.1 English frequency

The main effect of English frequency was the reverse of what is typically observed in wordlikeness experiments. Here, higher ratings were given to words containing low English frequency consonant sequences. The same result was observed by Richtsmeier (2011), who attributed the effect to the instructions: Participants were asked to make their ratings with respect to Martian, rather than to English. Participants in the present study also made their ratings with respect to Martian. In this context, expectations based on English may flip, and the lower the frequency of a target sequence in English, the more plausible the accompanying test wordform may seem as an instance of Martian.

The effect also presents an opportunity to examine experimental language manipulations apart from the influence of a participant’s native language. Here, the effects of English and experimental frequencies go in opposite directions (high experimental frequencies raise ratings, high English frequencies lower them), so a researcher interested in the learnability of a particular phonetic or phonological property may be able to manipulate that property in the experimental context of Martian and be confident that the results need not be attributed to some uncontrolled property of English.

### 4.2 Experimental frequency

Extraction of the target consonant sequences was inferred from the experimental frequency factor, that is, from a difference in ratings following exposure to one versus four familiarization words. Participants gave consistently higher ratings to words that contained high experimental frequency sequences, indicating a sensitivity to the relative frequencies of consonant patterns. Similar findings have been reported by Finley (2013), Goldrick and Larson (2008), Peperkamp and Dupoux (2007), Richtsmeier (2011), and others. If we assume that extraction effects are indicative of more general phonotactic and phonological learning, the finding is in line with previous studies of input-based phonological learning (Edwards et al., 2004; Zamuner et al., 2004), studies which consistently report a learning advantage for frequent phonological forms.

The experimental frequency effect was dependent on English frequency, however, as an English frequency × experimental frequency interaction was observed. The interaction reflects a difference in magnitude, with experimental frequency having a more robust effect for high English frequency sequences. In a similar experiment, Richtsmeier (2011) reported that the experimental frequency effect was numerically—albeit nonsignificantly—larger for high English frequency sequences. It is possible that the experimental frequency effect involves some priming of previous knowledge (Goldinger, 1996), and high English frequency sequences receive greater priming support. Additional research is necessary to understand the role that pre-existing knowledge plays in experimental learning effects.

Perhaps most importantly, the experimental frequency effect allowed for exploration of the semantics and phonological variability factors. In other words, the main effect of experimental frequency was expected, and the goal was to observe how the effect might change depending on the amount of phonological variability across related familiarization words, or depending on the association of referents to wordforms.

### 4.3 Phonological variability

The experiment varied the degree of phonological similarity between familiarization words that shared a target sequence. The purpose was to explore whether phonological generalization was dependent on the degree of variability across related words. Phonological learning algorithms treat all word-types equivalently (Albright, 2009; Boersma, 2011; Hayes & Wilson, 2008), but perhaps type-based learning is most effective when the word-types are phonologically dissimilar. Assuming a role for phonological variability, the predicted result was an experimental frequency × phonological variability interaction, with more robust experimental frequency effects in the moderate and/or high variability conditions. No interaction was observed, however, and the results may be taken as support for the assumptions of the learning algorithms listed above, as well as for an explicit definition of word-types as purely phonological. Nevertheless, the effect sizes of experimental frequency at each level of phonological variability suggest that ratings may be influenced by phonological variability (low variability, *r* = .06; moderate variability, *r* = .15; high variability, *r* = .19). Furthermore, the direction of that trend indicates a preference for word lists with greater variability. Given this finding, as well as observed limitations on token-based generalization (Albright, 2009; Richtsmeier, 2011), it seems worthwhile to consider how the degree of acoustic change across words might influence phonological learning.

**Figure 6:**
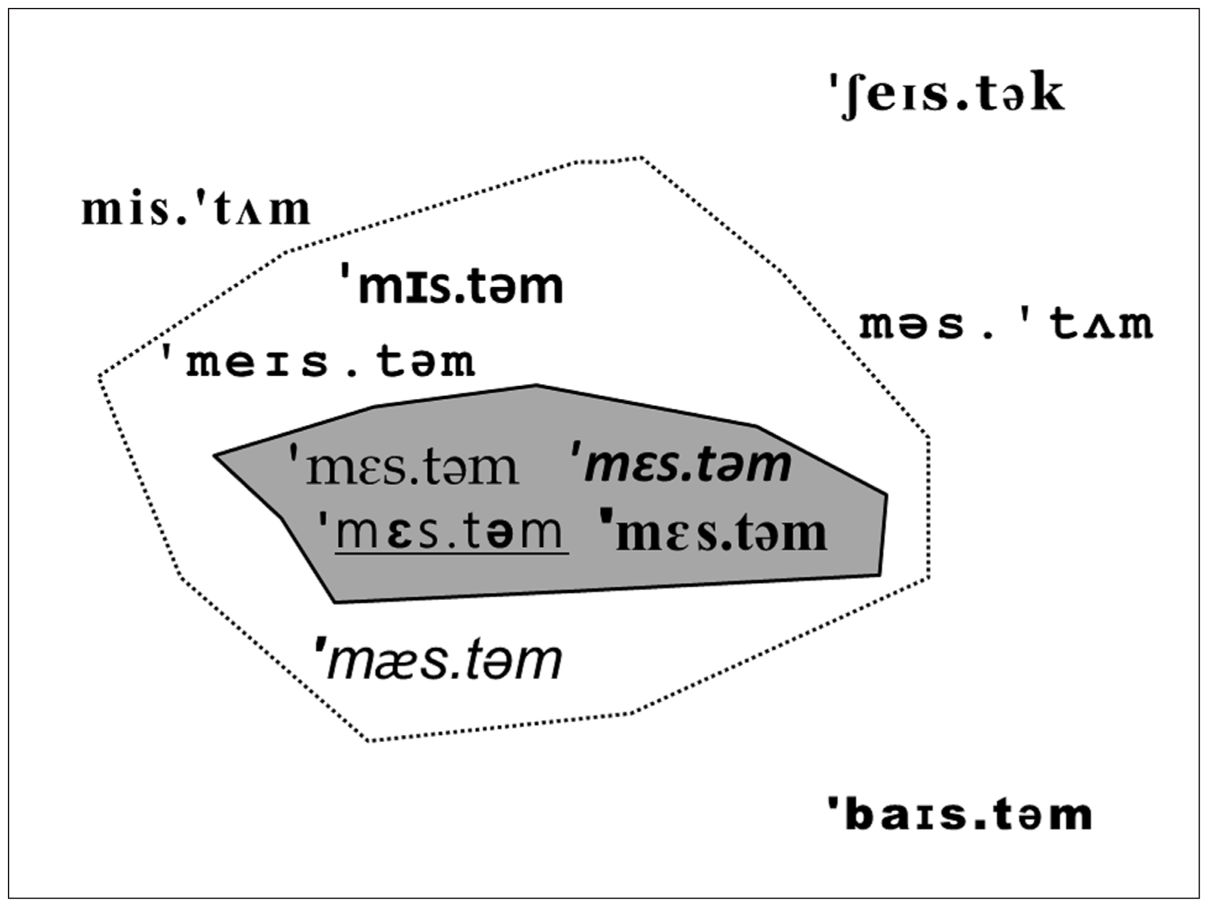
A sketch of hypothetical acoustic space populated by a set of words containing /st/. The shaded innermost ring represents the acoustic space occupied by the word-type /ˈmεs.təm/, with different fonts representing acoustic differences such as speaker variability. The next ring represents phonological changes limited to the first vowel, as in the low variability list. The outermost space is populated by the more varied word-types from the moderate and high variability lists.

If we think of phonology as dividing up an acoustic space populated by words, then the present experiments sketch out borders like those illustrated in Figure 6. The figure contains three areas separated by two concentric borders. The shaded innermost area represents the acoustic space occupied by a single word-type, in this case, /mɛstəm/, as well as the acoustic differences related to token variability, including changes in talkers. To date, most evidence suggests that token variability does not by itself support phonological learning (but cf. Richtsmeier et al., 2011, for evidence that the word-types and talker variability combine to support phonotactic learning in children).

Surrounding /ˈmɛs.təm/ is another border for the minimal phonological changes that result in new word-types, including /ˈmeɪs.təm/, /ˈmɪs.təm/, and /ˈmæs.təm/. This border is supported by the finding that speakers of a variety of languages treat consonants as more integral to a word’s makeup than the word’s vowels (Cutler et al., 2000). Finally, the outermost area includes the more substantial acoustic differences between word-types. These types include wordforms with different stress patterns like /mis.ˈtʌm/ and /məs.ˈtʌm/ from the moderate variability word list, as well as wordforms with different consonants like /ˈʃeɪs.tək/ and /ˈbaɪs.təm/ from the high variability list. The outermost area corresponds to the consistent learnability of word-types found here and in other studies. The findings leave several properties of these borders unresolved. For example, the acoustic border between types and tokens was not addressed because the phonological variability condition only considered changes at the phonemic level. Additionally, based on the increasing effect sizes for experimental frequency with great degrees of phonological variability, it is not clear how sharp the larger, dashed border is. Thus, one possible interpretation of the results is that a variety of phonemic sequences, rather than a variety of word-types, facilitated learning.

The interaction of phonological variability and English frequency should also be considered briefly. There were significant and large effects of English frequency for the low and moderate variability lists, but only a trend for the high variability list. The effects were all in the same general direction, however, and so it seems reasonable to favor interpretation of the main effect of English frequency. It is also unclear why English frequency effects might depend on phonological variability, so future research is required to better understand this finding.

### 4.4 Semantics

The semantics factor was the primary factor of interest in this study. Underpinning the manipulation of referents to wordforms was a question about the quiddity of word-types, and whether phonological learning might be sensitive to semantic cues. As with phonological variability, the predicted effect was an interaction of experimental frequency and semantics, with a stronger experimental frequency effect in the multiple referents condition compared to the single referent condition.

In contrast to expectations, experimental frequency and semantics did not interact in the main analysis. Furthermore, planned analyses at each level of phonological variability revealed consistent effects of experimental frequency but no interaction with semantics. Participants appeared to learn from frequent word-types, regardless of how meaning was assigned. Again, the results favor the existing definition of word-types as purely phonological entities. However, it is still possible that semantics may be sufficient for promoting generalization. This possibility could be explored in a study where multiple referents are assigned to the same wordform, that is, four separate referents assigned to different tokens of /mɛstəm/.

The main effect of semantics is noteworthy. Participants responded with significantly higher ratings when multiple words were associated with the same Martian animal. One possible explanation for the effect is that it reflects an intuition about the general permissibility of variation. In the single referent condition, participants were exposed to phonological variability for the same referent, resulting in high intrareferent variability. This may have led the single referent group to conclude that Martian generally allows a high degree of variation, making all test wordforms more plausible. In contrast, acoustic variation was closely aligned with referent variation in the multiple referents condition, and there was less intra-referent variation. This may have restrained participants’ intuitions about the Martian language’s permissiveness of variability, and thereby resulted in lower ratings for the same novel test wordforms.

#### 4.4.1 Relationship to previous findings linking semantics to phonetics and phonology

The findings contrast with previous work, although the differences were not completely unexpected. Peperkamp and Dupoux (2007) found that participants in a statistical learning study were better able to learn an allophonic alternation when the two related forms were consistently accompanied by referents. It is possible that semantics helped participants in that study grasp the importance of determiners for predicting the allophonic alternation, so semantics may act as a cue for learning morphological or syntactic patterns, which in turn act as cues for phonological patterns. It is also worth noting that Peperkamp and Dupoux did not observe phonological generalization across alternating segments, and it is possible that more robust generalization of an allophonic alternation depends on more robust phonological cues.

Yeung and Werker (2009) found that infants are better able to learn nonnative contrasts when infants are given semantic referents for each of the novel sounds, suggesting that semantics may act as a cue to phonological contrast. Similarly, Creel (2012) found that children interpreted phonologically odd wordforms like *feesh* as real words like *fish*, suggesting that existing representations bias how acoustic or phonological variation is interpreted. Semantic cues may be important for interpreting sounds near phonological borders, but it is not yet clear how this kind of disambiguation might influence type-based learning. Perhaps the clearest takeaway is that studies of phonological contrast do not allow straightforward comparisons to studies of phonological generalization. Furthermore, the relationship between phonological learning and semantics may be more complex than all or nothing, and future research may be able to better delineate when and where semantics matters to phonology. Allophony and phonological contrast are two areas where such a relationship may exist.

### 4.5 Limitations of the present study

In any statistical learning study with adults, there is a possibility that participants may engage in explicit learning of the target patterns, rather than the implicit learning that is typically assumed. There is a longstanding and continually growing literature on the differences between implicit and explicit learning, including in the area of human development (e.g., Rovee-Collier et al., 2001). Unfortunately, participants were not questioned following the experiment about what they may have learned explicitly. Explicit awareness of the targets may have been possible for some of the participants, as well as for participants in the Peperkamp and Dupoux (2007) and Creel (2012) studies. As such, future studies should probe for explicit learning, for example, during debriefing.

Four other limitations of the study should be noted. First, the results reflect learning patterns for adults, who may not learn from the same data that children learn from (cf. Richtsmeier, 2011, pp. 173–174). Thus, research with children can help to assess whether the present claims are true of language acquisition more generally.

Second, the present experiments used between-subjects manipulations of both phonological variability and semantics. All the claims made here should hold for a within-subjects design, which would allow for a stronger claim about the relative learnability of phonological and semantic cues to word-type status, but such a claim awaits empirical validation.

Third, although the study was intentionally designed to favor type-based learning, it does not address other potential means of phonological learning, for example, token-based learning. One potential solution to this problem would be to compare conditions with equal token frequency and different levels of type frequency. Participants in a high-type condition could be exposed to /st/ in 4 tokens each of four word-types; participants in a low-type condition could be exposed to /st/ in 16 tokens of one word-type. This design would result in an equal number of word-tokens across experimental frequency conditions and represents a more powerful method for assessing the relative learnability of word-types and word-tokens.

Fourth, the predicted effect of semantics rested on the assumption that participants would treat varying wordforms with the same referent as varying pronunciations of the same word. It is not possible to verify that participants actually drew that conclusion, however. As such, it is possible that semantics could influence type-based learning under conditions in which participants verifiably treated the familiarization words as varying pronunciations of the same word. Given this limitation, the results imply limitations to semantics in type-based learning, but they do not rule out a semantic influence entirely.

## 5 Conclusion

This experiment measured how participants learned phonotactic patterns from a brief familiarization. Phonological and semantic properties of the familiarization word sets varied, but it was only a basic type-frequency manipulation that consistently influenced learning. Thus, the results support the view that word-types are defined by their phonological properties.

## Figures and Tables

**Table 1:** A summary of the four experimental factors.

Factor	Description
1. English frequency	A within-subjects manipulation of the English frequency of the target word-medial consonant sequences. Factor levels were high (frequent in English) and low (infrequent in English).
2. Experimental frequency	A within-subjects manipulation of the number of familiarization words containing the target word-medial consonant sequences. Factor levels were high (four words) and low (one word).
3. Phonological variability	A between-subjects manipulation of how similar related familiarization words, or words containing the same word-medial consonant sequences, were to each other. Factor levels were low, moderate, and high variability.
4. Semantics	A between-subjects manipulation of whether related familiarization words, or words containing the same word-medial consonant sequences, had different visual referents or shared the same visual referent. Factor levels were multiple referents and single referent.

**Table 2:** The low variability familiarization word list. The two sequences that share a word frame appear in the same column (e.g., /fp/ and /st/).

		/fp/	/mk/	/pk/	/ʃp/
Low English frequency	Low experimental frequency	ˈmɪf.pəm	ˈseɪm.kəʃ	ˈbup.kəs	ˈfʌʃ.pət
	High experimental frequency	ˈmɪf.pəm	ˈsɪm.kəʃ	ˈbup.kəs	ˈfuʃ.pət
		ˈmeɪf.pəm	ˈseɪm.kəʃ	ˈbʌp.kəs	ˈfʌʃ.pət
		ˈmεf.pəm	ˈsεm.kəʃ	ˈbap.kəs	ˈfaʃ.pət
		ˈmaf.pəm	ˈsam.kəʃ	ˈbaʊp.kəs	ˈfaʊʃ.pət
		**/st/**	**/mp/**	**/kt/**	**/sp/**

High English frequency	Low experimental frequency	ˈmɪs.təm	ˈseɪm.pəʃ	ˈbuk.təs	ˈfʌs.pət
	High experimental frequency	ˈmɪs.təm	ˈsɪm.pəʃ	ˈbuk.təs	ˈfus.pət
		ˈmeɪs.təm	ˈseɪm.pəʃ	ˈbʌk.təs	ˈfʌs.pət
		ˈmεs.təm	ˈsεm.pəʃ	ˈbak.təs	ˈfas.pət
		ˈmas.təm	ˈsam.pəʃ	ˈbaʊk.təs	ˈfaʊs.pət

**Table 3:** The test wordforms. These wordforms were rated by all participants.

	/fp/	/mk/	/pk/	/ʃp/
Low English frequency	ˈgʌf.pək	ˈkaʊm.kən	ˈzeɪp.kən	ˈzaʃ.pək
	ˈnaf.pək	ˈgum.kən	ˈlεp.kəf	ˈkeɪʃ.pəf
	ˈtaʊf.pən	ˈnʌm.kəf	ˈtɪp.kəf	ˈlεʃ.pən
	**/st/**	**/mp/**	**/kt/**	**/sp/**

High English frequency	ˈgʌs.tək	ˈkaʊm.pən	ˈzeɪk.tən	ˈzas.pək
	ˈnas.tək	ˈgum.pən	ˈlεk.təf	ˈkeɪs.pəf
	ˈtaʊs.tən	ˈnʌm.pəf	ˈtɪk.təf	ˈlεs.pən

**Table 4:** Four orderings of the target sequences by block and experimental frequency. High English frequency sequences are in bold typeface. These orderings applied to all three familiarization word lists, and they were duplicated for the semantics factor: one for the single referent condition and one for the multiple referent condition. Note that both familiarization and test trials were randomized, which cannot be seen in this table.

		Ordering 1	Ordering 2	Ordering 3	Ordering 4
Block 1	High experimental frequency	**st**	fp	mk	**mp**
		**kt**	pk	ʃp	**sp**
	Low experimental frequency	mk	**mp**	**st**	fp
		ʃp	**sp**	**kt**	pk
Block 2	High experimental frequency	fp	**st**	**mp**	mk
		pk	**kt**	**sp**	ʃp
	Low experimental frequency	**mp**	mk	fp	**st**
		**sp**	ʃp	pk	**kt**

## Footnotes

1 One familiarization word from the low variability list, ˈbʌp.kəs, is a word borrowed from Yiddish meaning “nothing”, as in, “That blowhard knows bupkes.” Use of a real word in the experiment was inadvertent, and no participant reported noticing the presence of a real word during the experiment.
